# Morphology Engineering of the Asymmetric PS-*b*-P4VP Block Copolymer: From Porous to Nanodot Oxide Structures

**DOI:** 10.1021/acsapm.3c02120

**Published:** 2023-11-02

**Authors:** Sajan Singh, Tandra Ghoshal, Nadezda Prochukhan, Alberto Alvarez Fernandez, Jhonattan Frank Baez Vasquez, Pravind Yadav, Sibu C. Padmanabhan, Michael A. Morris

**Affiliations:** AMBER Research Centre and School of Chemistry, Trinity College Dublin, Dublin 2 D02AK60, Ireland

**Keywords:** block copolymer, metal oxide, self-assembly, inverse, solvent vapor annealing

## Abstract

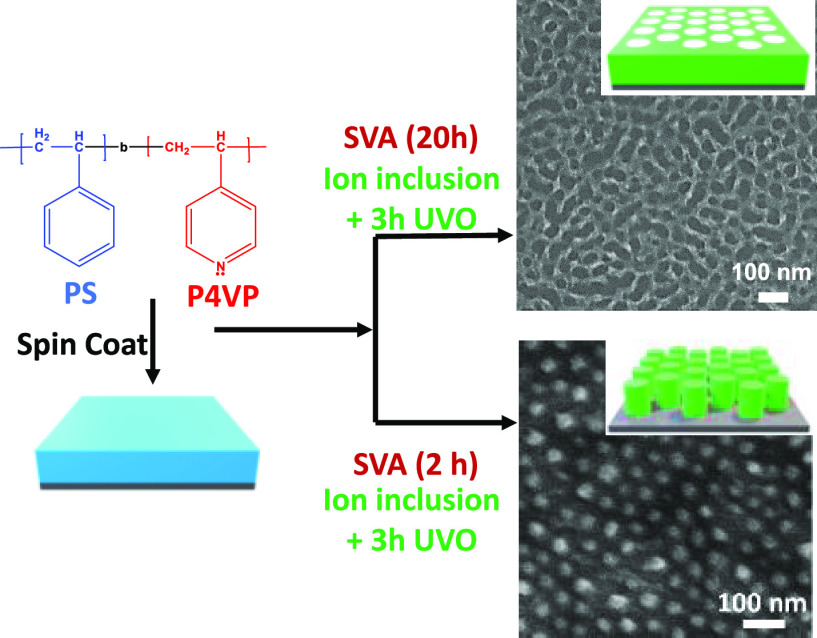

In the present work, we demonstrate the formation of oxide porous and nanodot structures from the same block copolymer (BCP) by the phase inversion of a BCP template. We investigated the effect of solvent annealing time on the ordering of asymmetric, cylinder forming, polystyrene-*b*-poly(4-vinylpyridine) (PS-*b*-P4VP) BCP. Phase separation of PS-*b*-P4VP was achieved by solvent vapor annealing (SVA) in a solvent atmosphere that is (partially) selective to P4VP to initially generate hexagonally arranged, cylindrical arrays of the expected structure. The morphology of the BCP changed from P4VP hexagonally packed cylinders to an 'inverse’ structure with PS cylinders embedded in a P4VP matrix. This suggests that selective swelling occurs over time such that the swollen P4VP phase becomes the majority volume component. Metal ions (Ga^3+^, In^3+^) were infiltrated into the BCP templates by a solution-mediated infiltration approach, followed by an ultraviolet-ozone treatment to remove the polymer and oxidize the metallic ions to their oxides. The findings show that a single BCP can be used to create both metal oxide arrays and porous structures of metal oxides by simply varying the duration of the solvent annealing process. The resulting structures were analyzed through several methods including scanning electron microscopy, atomic force microscopy, X-ray photoelectron spectroscopy (XPS), transmission electron microscopy, and energy-dispersive X-ray spectroscopy. XPS analyses confirmed the complete elimination of the BCP template and the presence of metal oxides. This study provides important insights into the development of functional BCP materials with inverse structures.

## Introduction

The fabrication of closely packed inorganic structures is pivotal in various fields like optoelectronics, photonics, sensors, field emission, catalysis, membranes, energy conversion, energy storage devices, and nanomedicine.^1−14^ Research on block copolymers (BCPs) has been driven by their potential application in nanotransistor fabrication because of their high periodicity, feature size and precise size.^13,15^ Self-assembly using BCPs is now widely accepted as an alternative or complementary approach to traditional photolithography, enabling the creation of microdomain arrays in a thin film form with a range of nanoscale feature sizes (5–100 nm) and versatile morphologies.^16−20^ However, these systems have many other potential applications as summarized by us elsewhere.^21,22^ For example, Cian et al.^21^ provided a comprehensive overview of diverse applications employing BCP templates across multiple domains, encompassing areas such as light-harvesting (energy), metasurfaces (photonics), nanofiltration membranes (environmental), and antibacterial activity (biological). In another review article,^22^ the authors emphasized the strategies that have emerged in BCP film formation to integrate inorganic materials. These films serve as on-chip etch masks for advancing next-generation electronic devices. Furthermore, as also demonstrated in these studies, the precise control of the BCP processing conditions is crucial in achieving the desired orientation of the microdomains for the formation of microphase-separated structures. Building on this understanding, it is essential to recognize that the self-assembly of BCPs depends on the Flory–Huggins interaction parameter (χ), which “measures” the chemical dissimilarity of the blocks and the degree of polymerization (N). Precision in controlling the microphase separation and self-assembly can be achieved by adjusting process parameters such as molecular weight, block volume fraction, block chemistry, and surface energy.^23^ This level of control has been pivotal in tailoring BCP thin films for a wide array of applications as previously explored.

The most convenient way to create thin polymer films on flat substrates is by spin-coating a dilute solution. However, the resulting BCP films are often not in equilibrium states since microphase separation and vitrification compete during solvent evaporation.^24,25^ To overcome this, the films can be self-assembled by annealing the BCP thin film under suitable conditions. This can be achieved through thermal annealing at elevated temperatures or by using solvent vapor annealing (SVA) at close to room temperature. SVA can produce distinct film patterns at much lower process temperatures and times than those with thermal annealing. In solvent annealing, the BCP film is exposed to a specifically selected solvent vapor environment. These vapors can preferentially expand the polymer blocks, effectively lowering the glass transition temperature (*T*_g_). As a result, this reduction in *T*_g_ enhances the mobility of the polymer chains and promotes better ordering within the structure.^26−29^ The BCP can also act as a designed template for the incorporation of inorganic materials. By selectively removing one copolymer block or introducing certain chemicals to a specific block, diverse material designs can be achieved through additional processing.^30−32^ The use of BCPs to form oxide arrangements have significant potential with properties like magnetism, ferroelectricity, semiconductivity and superconductivity.^33^ These BCP-derived oxide patterns are usually in the form of lines or dots (by infiltration of lamellar forming or hexagonal forming BCPs).^19,34,35^ For example, Ghosal et al.^19^ from our research group recently demonstrated a range of sub-25 nm well-ordered inorganic and dielectric nanodots and nanowire array patterns on the substrates using the BCP template. Here, they have used a cylindrical-phase poly(ethylene oxide)-*b*-polystyrene (PS-*b*-PEO) BCP as a template with hexagonally ordered perpendicular or parallel orientation of PEO cylinders. Moreover, in another study,^35^ they created the high aspect vertical pore arrangements on a substrate. However, to achieve the porous pattern, they used a microphase separated PEO-*b*-PS BCP thin film, where PS, not PEO, is the cylinder forming phase and PEO is the majority block. In a study carried out by Esmeraldo Paiva et al.,^13^ they also demonstrated the fabrication of vertically aligned pores on the substrate using P2VP-*b*-PS BCP, where again PS, not P2VP is the cylinder forming phase and P2VP is the majority block. However, forming continuous oxide films with vertical pores is less well demonstrated despite their potential use in, for example, sensor and diagnostic tests because of their high surface area and size selectivity. To the best of our knowledge, the specific formation of both porous and nanodot structures using a single PS-*b*-P4VP self-assembled BCP via a selective solvent approach has not been reported thus far.

This work is a systematic investigation of the effect of solvent annealing time on the ordering of highly asymmetric, cylinder-forming polystyrene-*b*-poly(4-vinylpyridine) (PS-*b*-P4VP) BCP (PS is the majority phase), as a route to oxide surface nanoarrangements. The BCP structures were produced through the microphase separation of a thin film exposed to a P4VP selective solvent, chloroform. The solvent initially induced the formation of a hexagonally close packed (HCP) cylinder structure arrangement with cylinders perpendicular to the substrate. However, the morphology of the BCP changed from P4VP hexagonally packed cylinders to an 'inverse’ structure with PS cylinders embedded in a P4VP matrix by extending the solvent annealing time. This provides a unique approach to generate inorganic films with either nanodot or nanopore structures from the same BCP. The oxide structures were fabricated using metal ions (Ga^3+^, In^3+^) selectively infiltrated into the BCP template via a solution-mediated method, taking advantage of the nitrogen lone pair in P4VP. The presence of the metallic ions in the BCP structure is confirmed by EDX. A subsequent ultraviolet-ozone (UVO) treatment was used to produce the corresponding metal oxide structures/patterns by oxidizing the metal and anchoring them onto the substrate while eliminating the BCP template. The oxidation of the corresponding metallic ions is corroborated by the XPS results. As a result, highly ordered metal-oxide nanodot arrays and porous structures were fabricated, opening possibilities for their use in optoelectronics, nanofabrication, membrane science, sensors, and catalysis. This research sheds light on the development of functional BCP materials, specifically with inverse structures.

## Experimental Section

### Materials

The cylinder forming PS-*b*-P4VP BCP was procured from Polymer Source and used without further purification (number-average molecular weight, Mn, PS = 40.5 kg mol^–1^, Mn, P4VP = 16.5 kg mol^–1^, polydispersity was 1.03). Silicon (100) wafers with a native oxide layer were used as a substrate. These were cleaned by ultrasonication in acetone and toluene for 20 min in each solvent and dried with N_2_ gas. Gallium(III) nitrate hydrate, indium(III) nitrate hydrate, chloroform, toluene, tetrahydrofuran (THF), and ethanol (dehydrated) were procured from Sigma-Aldrich and used without further purification.

### Fabrication of Block Copolymer Nanopatterns and Inclusion of Metal-Oxide

**Figure 1:**
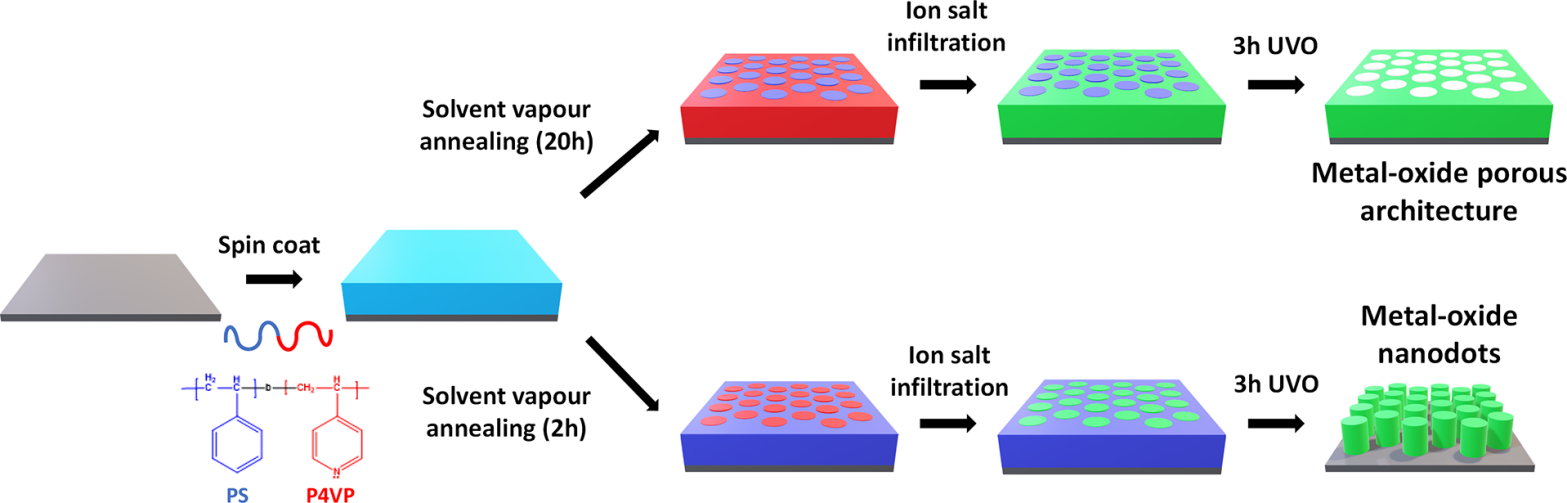
Schematic representation for Fabrication of metal oxide arrays and porous structure via a block copolymer template. Green color shows the metal ion.

PS-*b*-P4VP BCP was dissolved in THF to yield 1 wt % polymer solution by stirring overnight at room temperature. The PS-*b*-P4VP thin films were prepared by spin coating at a speed of 3000 rpm for 30 s with a ramp of 5 s. The SVA process was conducted as follows: the as-spun film on substrates was placed in a 250 mL (75 × 70 mm) glass jar completely closed with a vial (50 mm × 25 mm) containing 3 mL of CHCl_3_ for a different period of time (30 min to 20 h) and at 5 ± 1 °C (maintained through the temperature-controlled refrigerator) to induce microphase separation over the required chain mobility. The vapor pressure for chloroform at 5 °C was 101 mbar, calculated using the Antoine Equation (see Supporting Information, Table S2). The samples were then removed from the jars at the specific desired times and allowed to stand in air at room temperature for trapped solvents to evaporate. For the fabrication of metal oxide nanodots, the respective metal salt precursor was dissolved in ethanol (0.25% w/w) and spin-coated onto the BCP films obtained earlier. The concentration was chosen to ensure that we filled the features completely while avoiding any excess deposition on the film’s surface. The BCP thin film, now infiltrated with metal cations, was washed with ethanol to remove any residual unreacted salt. The film was then gently dried using a nitrogen flow. Following this, UVO treatment for 3 h was employed to achieve both oxidation of the precursor and removal of the BCP template. The mechanism by which UVO eliminates the BCP template is now well-established.^36^ In UVO treatment, ozone, an active oxidizing agent, is generated in situ from atmospheric oxygen by exposure to 185 nm of UV light. The ozone produced subsequently photosidicates into molecular oxygen and atomic oxygen upon exposure to 254 nm light. The latter species react with the polymer to form free radicals and activated species that eventually remove organic portions of the polymer in the form of carbon dioxide, water, and a small amount of volatile organic compounds. This treatment utilized a PSD Pro Series Digital UV Ozone System manufactured by Novascan Technologies, Inc., based in the USA. A schematic for the fabrication of metal-oxide arrays via block copolymer template is shown in Figure 1.

### Characterization

Atomic force microscopy (AFM) (Park Systems, XE7) and scanning electron microscopy (SEM) (FEI Company, FEG Quanta 6700, and Zeiss Ultra Plus) were used to analyze the surface morphologies. The AFM has a silicon microcantilever probe tip and an ambient force constant of 42 N/m, AFM was run in the noncontact mode. An FEI Helios Nanolab 600i system with a high-resolution Elstar Schottky field-emission SEM and a Sidewinder FIB column was used to prepare samples for TEM cross-sectional imaging. On the FEI Titan, TEM and elemental mapping were performed. SEM cross sections were achieved from samples cut in half and placed perpendicular to the incident electron beam with a tilt angle of 20° (70° to the incident beam). The film thicknesses were measured by an optical ellipsometer (Woolam M2000) and electron microscopy.

XPS (VG Scientific ESCAlab Mk II) was carried out under an ultrahigh vacuum (5 × 10^–10^ mbar) with a hemispherical analyzer and Al K X-rays (1486.6 eV). The photoelectrons released were collected at a 90° angle from the sample surface. The analyzer pass energies for survey scans were set at 100 and 40 eV for high-resolution core scans. A binding energy of 284.8 eV for a C 1s signal was used as the internal calibration.

## Results and Discussion

### Self-Assembly by Solvent Annealing

**Figure 2:**
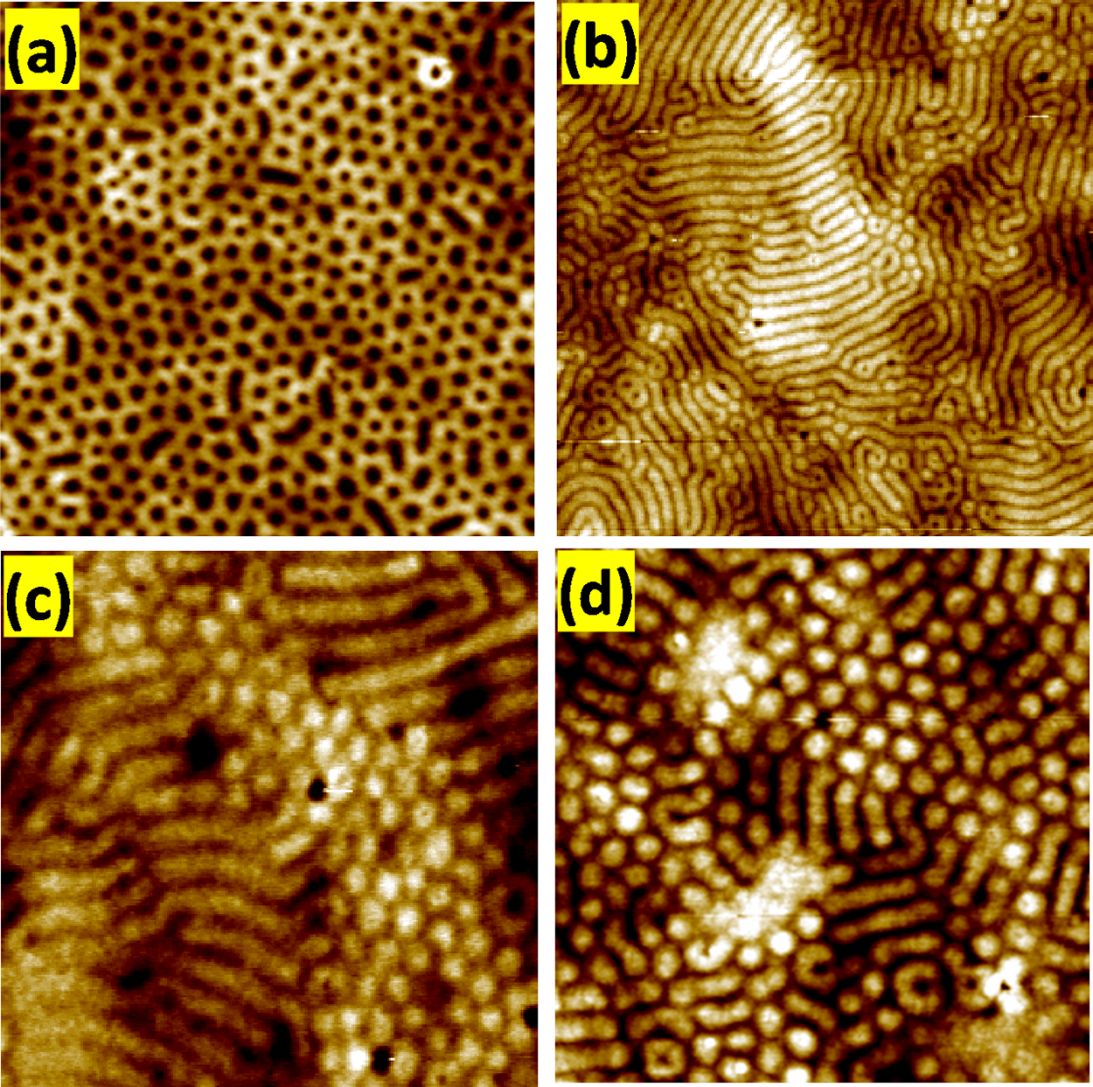
AFM micrographs (1 × 1 μm^2^) of PS-*b*-P4VP (40.5-*b*-16.5) kg mol^–1^ after solvent annealing at 5 °C for different annealing times; (a) 2 h, (b) 4 h, (c) 15 h, and (d) 20 h.

Note that solvent vapor annealing at low temperatures (e.g., 5 °C) has advantages such as a slower evaporation rate, which allows for more uniform solvent distribution and better control over the degree of swelling, lowering the risk of defects and other unfavorable morphological changes. Additionally, the lower temperature used ensures that the polymer mobility in the solvent annealing experiment is restricted. This is important for generating higher quality films (i.e., block morphologies and dewetting) in this work. Solvent annealing can be difficult to control, because polymer mobility can increase rapidly as it swells. It is also important to point out that solvent inclusion dramatically changes phase diagrams. Because of this, great care is needed to ensure that the swelling can be controlled enough to allow the various morphology changes to be properly controlled avoiding more random and poorly ordered films.^13,29,37−40^ Chloroform was chosen as the solvent because it can swell both blocks but has preferential affinity for the minority P4VP block. Experimental evidence has also shown chloroform’s preference for interacting with the P4VP block.^41^ Additionally, the Hansen solubility parameter differences between the solvent and both blocks (see the Supporting Information) suggest significant preference of chloroform with PV4P compared to PS. Because it can swell both blocks, it was considered a good choice as the vapor phase would be block neutral preventing preferential segregation of one block and this favoring vertical (to the surface plane) oreientation of cylinders. The morphological changes that occurred during annealing were captured using noncontact mode topographical AFM at various exposure times, as depicted in Figure 2. The AFM images captured over a large area indicate that all four samples displayed microphase-separated structures and did not experience significant film dewetting. The film thickness of the polymeric samples was investigated by spectroscopic ellipsometry. Thus, no appreciable changes in thickness have been observed after the SVA process, obtaining in all cases film thickness around 100 ± 3 nm (Supporting Information, Figure S1A,B). Notably, the morphologies observed changed over time; for the sample annealed for 2 h, vertically aligned cylinders (resembling dots) were visible, with an average center-to-center distance of 67 ± 5 nm and a diameter of 27 ± 4 nm. As the annealing period increased, the morphology transitioned from dot structures to fingerprint patterns suggesting a change of phase from hexagonal to lamellar. Interestingly, we observed a morphology resembling the formation of dots again after a 20 h annealing period. However, the average diameter (48 ± 5 nm) and center-to-center distance (49 ± 3 nm) of the cylindrical domain were found to be different from those obtained after a 2 h annealing time. We suggest that this is because the morphology with hexagonally packed P4VP cylinders was transformed into an “inverse” morphology with PS cylinders embedded in a P4VP matrix, as will be confirmed below.

We propose that the observed transition from P4VP cylinders in the PS matrix to PS cylinders in P4VP matrix is likely due to the preferential affinity of chloroform for the P4VP block, which leads to significantly greater swelling of the P4VP block than the PS block. This is supported by comparison of the Hansen Solvent Parameters which show that chloroform has a significantly higher affinity for the PV4P block compared to PS (see Supporting Information). Polymer swelling is a diffusion process determined by a number of factors, including chemical similarity (solubility parameters), solvent concentration, molecular sizes, temperature, chain packing, and glass transition temperature. Relating swelling to changes in a system under solvent exposure is therefore complex. We believe that both blocks are at least partially swollen in these conditions on the basis of the calculated interaction parameters (see the Supporting Information). However, we believe that the P4VP block swells considerably more than PS so that the effective volume of the P4VP block becomes close to that of the PS block and then swells further to form a majority volume in the BCP film. In this way, a transition to a lamellar pattern first results, as observed here. Swelling continues until the P4VP has a significantly greater volume, and the inverse hexagonal phase is formed. Direct explanation based on solvent parameters is a appropriate because both polymers have similar molecular structures and crystallinity^29,41^ and although their glass transition temperatures differ by 50 °C, they are both >100 °C and might be expected to reach swelling equilibrium in these conditions.^42,43^ This hypothesis is consistent with previous studies that showed the volume ratio of the blocks and interaction parameters to be a critical parameter in determining the self-assembly morphology of BCPs in solution and thin films.^44,45^ Our findings provide a better understanding of the factors that govern the morphological transition in BCP thin films during solvent annealing. The AFM image shows that the inverse structure began to appear after 15 h of solvent annealing.

### Selective Metal Infiltration

The inverse morphology was further corroborated by the selective inclusion of metal salt into the BCP templates. Ordered arrays of metal oxide can be created by infiltrating metal into a polymeric template and converting it to metal oxide through UVO. In order to achieve this, hexagonal close-packed PS-*b*-P4VP patterns that were obtained by annealing for 2 and 20 h (Figure 2a,d) were used as templates for infiltrating a precursor solution of gallium cations. Infiltration of the inorganic precursors on the BCP film was confirmed also by spectroscopy ellipsometry. While the total thickness of the film remains constant after infiltration (around 100 nm), the refractive index (*n*) of the hybrid film increases as a result of the selective incorporation of the metallic ions on the BCP domains. Thus, *n* values increase from 1.51 (corresponding to pure BCP films) to 1.81 after the inorganic precursor infiltration (Supporting Information, Figure S2A).

**Figure 3:**
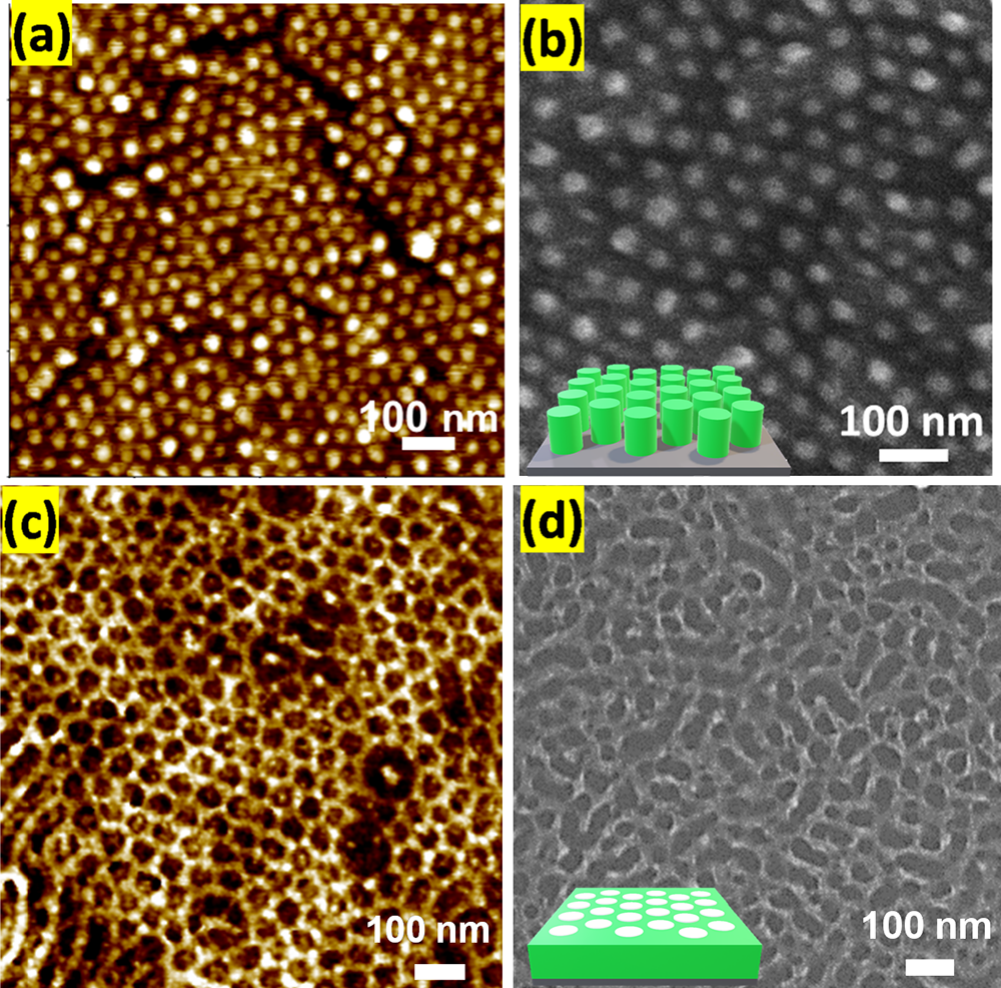
Topographical AFM and SEM images of gallium oxide patterns created using BCP templates (a, b) 2 h annealing and (c, d) 20 h annealing. Insets of figure (b) and (d) represent their corresponding schematic of the morphology.

In a subsequent step, BCP templates are removed by UVO treatment, at the same time inducing the formation of the corresponding inorganic oxides from the inorganic precursors. The resulting gallium oxide nanopatterns were imaged using AFM and SEM (Figure 3), formed by the spin coating of an ethanolic gallium nitrate solution onto the polymer templates, followed by UVO treatment. It is noteworthy that the metal oxide patterns resulting from the BCP templates exhibited distinct variations. For the BCP template (obtained at 2 h annealing), Ga nitrate penetrates the cylindrical domain of the BCP template, leading to the formation of arrays of gallium oxide (as shown in Figure 3a) as expected. Moreover, the average diameter of metal oxide dots and center-to-center distances were found to be 30 ± 3 and 45 ± 3 nm, respectively. Conversely, for the BCP template (obtained at 20 h annealing), Ga nitrate penetrated the matrix of the BCP template, resulting in the development of a layered metal oxide structure with a porous appearance (as depicted in Figure 3c). Furthermore, the average diameter and center-to-center distance were determined to be 41 ± 3 and 61 ± 4 nm, respectively. Final thickness of the inorganic structures created on top of the silicon substrates was evaluated also by spectroscopic ellipsometry. In both cases, thicknesses of 7.2 ± 2 nm have been observed (Supporting Information, Figure S2B,C).

The decrease in pore diameter and increase in pore center-to-center distance also aid infiltration in the BCP matrix. The possible infiltration of gallium nitrate into the BCP matrix can be explained for two reasons. The first reason is the hydrophobic insertion, which involves the infiltration of Ga^3+^ into the PS matrix. However, this is an unfavorable condition since there are no functional groups available in the PS phase. The second reason is the formation of an “inverse” structure where PS cylinders are embedded in the P4VP matrix, and hence, Ga^3+^ is infiltrating the P4VP matrix. To further support the inverse morphology, we added another metal salt, indium nitrate (In^3+^) along with Ga^3+^, followed by UVO treatment. The AFM and SEM images were also recorded for the resulting pattern of mixed Ga and In oxide, as shown in the Supporting Information, Figure S3. Final thickness of the bimetallic structures was established at 8.2 ± 1 nm, confirming the successful infiltration and formation of the metallic structure (Figure S2D). We subsequently used TEM-EDX to analyze the resulting BCP-templated metal oxide nanopatterns.

**Figure 4:**
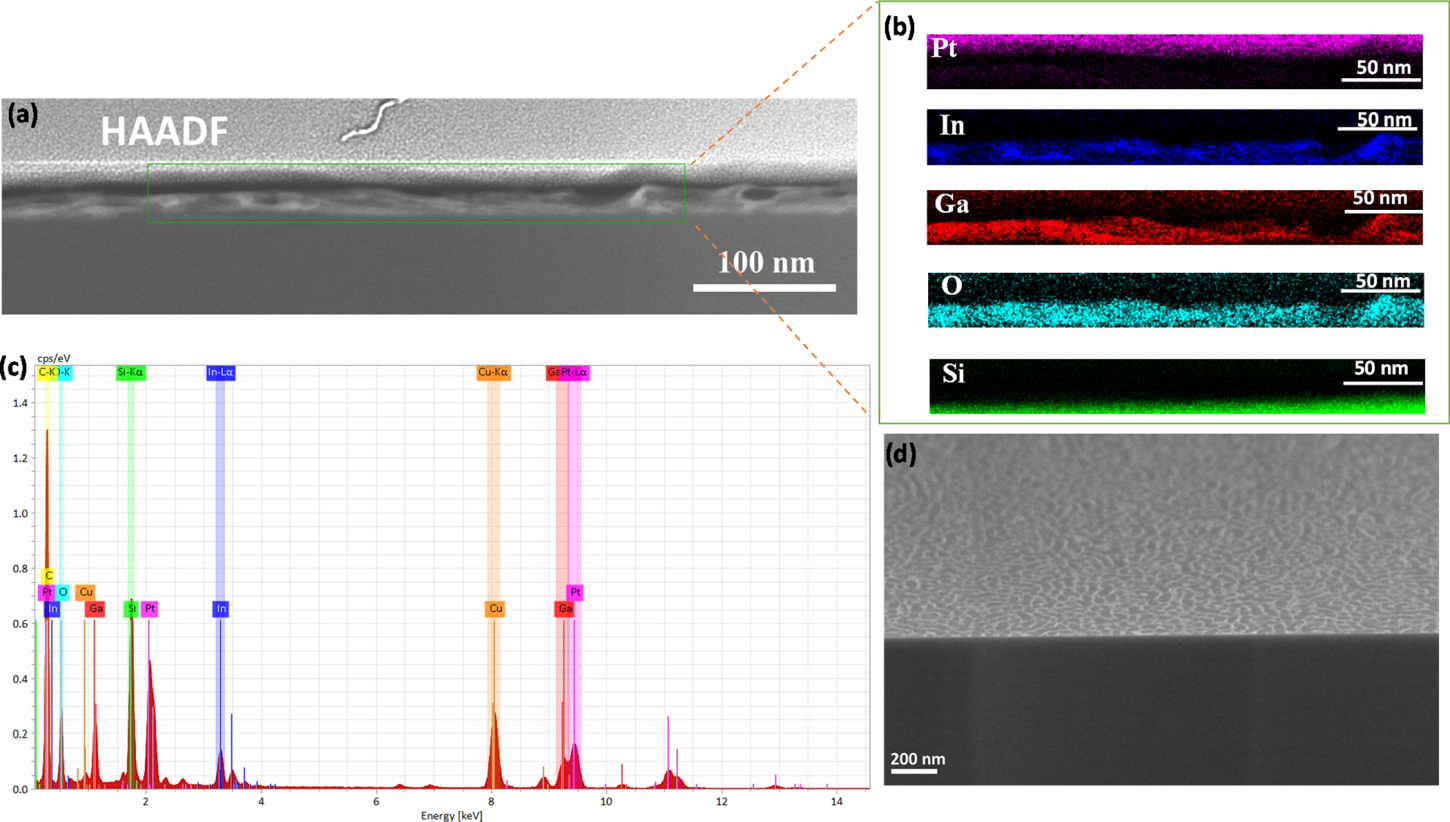
(a) TEM cross-sectional image detailing the porous appearance structure, (b) elemental mapping of Si, In, Ga, O, and Pt, (c) EDX analysis of corresponding elements, and (d) cross-sectional SEM image again displaying the porous structure.

Figure 4 displays a HAADF-STEM image and corresponding elemental maps for Ga, In, O, Si, and Pt, which demonstrate the locations of both gallium and indium in the BCP template. In Figure 4a, the metal oxide layer can be observed above the silicon oxide native layer in an undulated shape. Figure 4b displays the results of the EDX mapping that was used to confirm the presence of the elements. The In- and Ga-mapped images clearly show the presence of both metals at the same location. This unambiguously confirmed that the matrix is made of P4VP, and that both Ga^3+^ and In^3+^ are located exclusively in the matrix phase of the BCP template, thereby corroborating the inverse morphology at 20 h annealing time. Additionally, the EDX spectrum (Figure 4c) confirmed the presence of the elements involved in the process. Furthermore, as a complementary analysis, cross-sectional SEM was also conducted, and Figure 4d shows the micrograph of the Ga/In oxide layered structure. The SEM image revealed a high substrate coverage by the metal oxide with no significant defects. It must be noted that these two metal oxides were chosen here because of potential applications as antimicrobials (Ga) and semiconducting oxides (In). They are also highly practical because of good SEM imaging as heavy metals and ease of detection for EDAX.

**Figure 5:**
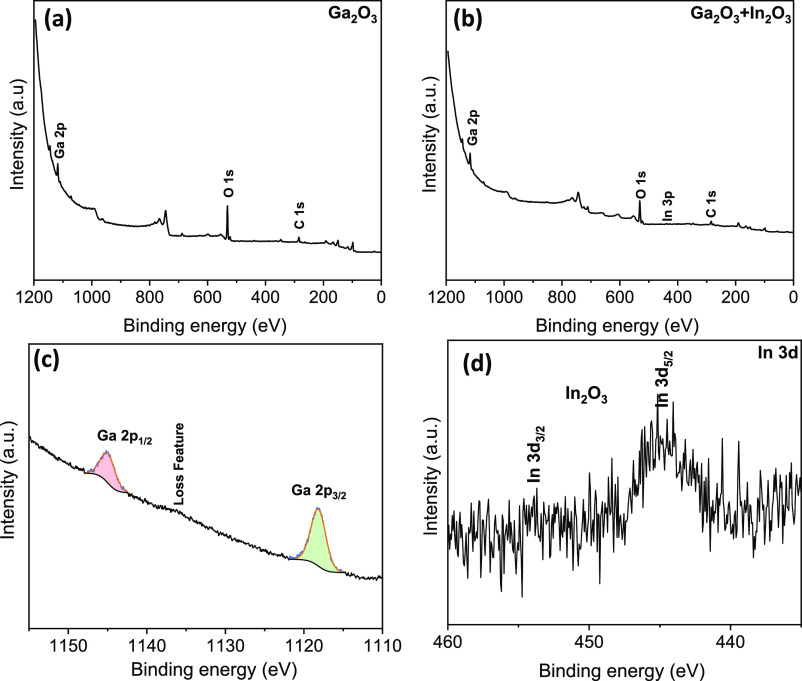
XPS spectra for the metal oxide obtained after 3 h UVO (a) Ga_2_O_3_, (b) Ga_2_O_3_ + In_2_O_3_, (c) Ga 2p, and (d) In 3d. The BCP template used here was formed after 20 h of solvent annealing.

To evaluate the metal infiltration and polymer removal after the UVO treatment, XPS analysis was performed. The BCP spectra in Figure S4a revealed peaks associated with carbon (C 1s), nitrogen (N 1s), and oxygen (O 1s), where the presence of oxygen was related to adventitious carbon species. Following the spin coating of Ga nitrate solutions, peaks associated with Gallium (Ga 2p) were observed alongside the carbon, nitrogen, and oxygen peaks (Figure S4b). The purpose of the UVO process was to eliminate the BCP template and convert the metal cations into metal oxides. The absence of a nitrogen peak in the XPS spectra (Figure 5a,b) of the UVO-treated samples confirmed the complete removal of the polymer. Furthermore, the peak for Indium was found to be very small, which is most likely due to the limited number of nitrogen atoms available for coordination with Indium subsequent to gallium nitrate infiltration. This provides additional evidence for the infiltration of Indium in the P4VP block and thus confirms the presence of both metals in the same location in the EDX mapping. In addition, high-resolution spectra were obtained for selected atomic orbitals of Ga and In from mixed Ga and In oxide samples, and these are displayed in Figure 5c,d. Figure 5c illustrates the Ga 2p XPS spectra, from the fitting process it is found that samples infiltrated with Ga(NO_3_)_3_ present peaks at 1118.1 and 1144.9 eV, corresponding to the Ga^3+^ 2p_3/2_ and Ga^3+^ 2p_1/2_ spin–orbit coupling components, respectively. The fitting parameters and obtained fitting values like peak area and fwhm have also been added in the Supporting Information, Table S3. These peaks, together with the one at 531.44 eV from the O 1s transition (Figure 5a) confirm the presence of Ga_2_O_3_ as the main surface compound left after PS-P4VP removal. Similarly, the XPS spectra for In 3d (Figure 5d) display two peaks at 444.8 and 454 eV, assigned to the In 3d_5/2_ and In 2p_3/2_ spin–orbit coupling components, respectively. Notably, the core-level scans of the metal peaks indicate the presence of oxides. The implications of these findings are significant for the advancement of functional BCP materials with inverse structures as well as their potential applications in optoelectronics, nanofabrication, and catalysis. These results also pave the way for further research in this field.

## Conclusions

In this study, we investigated the potential of creating morphology-engineered structures such as porous and nanodots using the same PS-*b*-P4VP BCP through preferential solvent assisted microphase separation and post processing. The phase separation was achieved via SVA in a solvent atmosphere that selectively favored P4VP, initially leading to the formation of hexagonally arranged, P4VP cylindrical arrays embedded in PS marix as expected. The morphology of the BCP changed from P4VP hexagonally packed cylinders to an 'inverse’ structure with PS cylinders embedded in a P4VP matrix by extending the solvent annealing time. This was confirmed by the selective infiltration of Ga and In cations into the respective BCP templates. The resulting Ga cation infiltration into the respective BCP templates demonstrated the creation of well-ordered nanodot arrays and porous structures of gallium oxide on the substrate with uniform diameters. The results demonstrate that a single BCP can be used to create both arrays and porous structures of metal oxides by simply varying the duration of the solvent annealing process. These findings have significant implications for the development of functional BCP materials with inverse structures and their potential applications in optoelectronics, nanofabrication, membrane science, nanosensors, and catalysis and open up possibilities for further research in this area.

## Supporting Information Available

Calculation for the interaction of monomer with solvent, ellipsometric thickness measurements, AFM and SEM image of mixed Ga and In oxide, and XPS spectra for BCP and BCP + Ga nitrate (PDF)
