# Supplementary material for: Morphology Engineering of the Asymmetric PS-b-P4VP Block Copolymer: From Porous to Nanodot Oxide Structures
Source: ACS Appl Polym Mater. 2023 Nov 2;5(11):9612–9. doi: 10.1021/acsapm.3c02120 (PMC10644307; doi:10.1021/acsapm.3c02120)
Supplement: Supplementary file 1 — ap3c02120_si_001.pdf [file ap3c02120_si_001.pdf]

Supporting Information

Morphology engineering of asymmetric PS-b-P4VP block copolymer: from porous to nanodots oxide structures

Sajan Singh*, Tandra Ghoshal, Nadezda Prochukhan, Alberto Alvarez Fernandez, Jhonattan Frank Baez Vasquez, Pravind Yadav, Sibu C. Padmanabhan, Michael A. Morris*

AMBER Research Centre and School of Chemistry, Trinity College Dublin, Dublin 2, D02AK60, Ireland

Correspondence*:* ssingh5@tcd.ie, morrism2@tcd.ie

Interactions for PS-P4VP using Hansen model:

The Hansen solubility model was used to estimate polymer-solvent interactions and the relevant parameters are represented in **Table S1**.[1,2,3]

Table S1 Hansen parameters for the species used in this study.

Material	δ_d	δ_p	δ_h
**Poly 4-vinylpyridine (P4VP) ***	18.1	7.2	6.8
**Polystyrene (PS)**	21.3	5.8	4.3
**Chloroform ($CHCl_3$)**	17.8	3.1	5.7
**Tetrahydrofuran (THF)**	16.8	5.7	8.0

The distance between the solvent and polymer points in the Hansen sphere (Ra) was calculated for PS/$CHCl_3$, P4VP/$CHCl_3$, PS/THF, P4VP/THF pairs, according to **Equation S1:**

$$(R_a)^2 = \left(4(\delta_{D1} - \delta_{D2})^2 + (\delta_{P1} - \delta_{P2})^2 + (\delta_{H1} - \delta_{H2})^2\right)\#[S1]$$

where δ_d, δ_p, δ_h represent the dispersion forces, permanent dipole-dipole forces, and hydrogen bonding as contributions respectively. Subscripts 1 and 2 denote the respective polymer and solvent species, respectively.

The interactions or distance Ra were calculated as follows: *Ra (P4VP-$CHCl_3$)* = 4.2, Ra (*PS-$CHCl_3$)* = 7.6, *Ra (P4VP-THF)* = 3.23, and *Ra (PS-THF)* = 9.7.

Based on practical consideration, solubility is generally observed for Ra values ≤ 8 MPa $^{1/2}$.[4] Therefore, we can confirm that chloroform is capable of solubilizing both PS and P4VP and is often considered as a good solvent both the blocks but having preferential interaction or affinity with P4VP blocks compared to PS blocks. Experimental evidence has also shown chloroform's preference for interacting with the P4VP block.[5]

* Hansen parameters belongs to the 4-vinylpyridine monomer, as experimentally determined values for P4VP were not found to the best of my efforts. Previous research also used the same values in case of P4VP.[6]

Table S2. Antoine Coefficients for Chloroform[7]

Temperature Range (°C)	A (mmHg)	B (mmHg)	C (mmHg)
0-78	4.207	1233.129	-40.953

$Log_{10}(P) = A-(B/(T+C)$

P= Vapor Pressure (bar)

T = temperature (K)

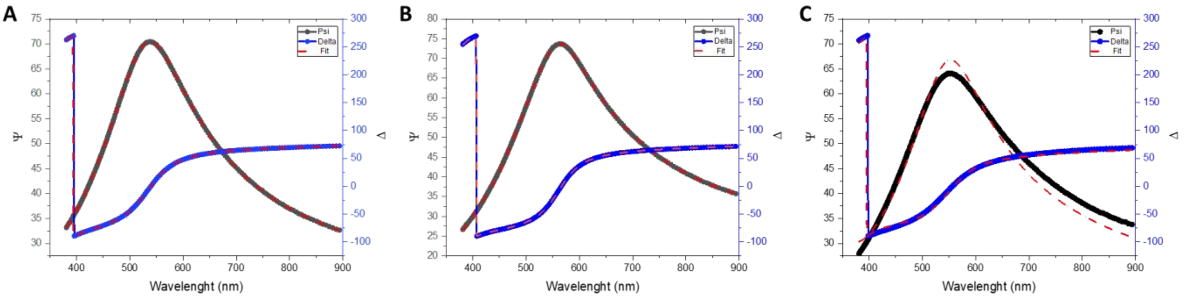

Figure S1. Evolution of the measured ellipsometric angles, Ψ (black) and Δ (blue), as a function of the wavelength for an angle of incidence of θ = 70° for A) BCP thin film after spin-coating, BCP thin film after 2h (B) and 20h (C) SVA treatment.

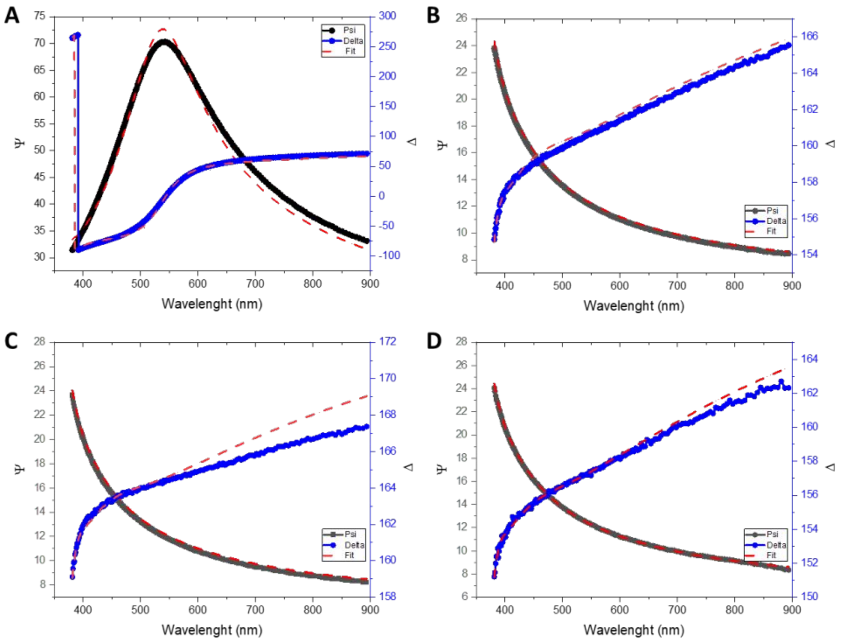

Figure S2. Evolution of the measured ellipsometric angles, Ψ (black) and Δ (blue), as a function of the wavelength for an angle of incidence of θ = 70° for A) BCP thin film after infiltration with Ga precursors and silicon substrate decorated with Ga (B), In (C) and Ga/In (D) oxide obtained after UVO treatment.

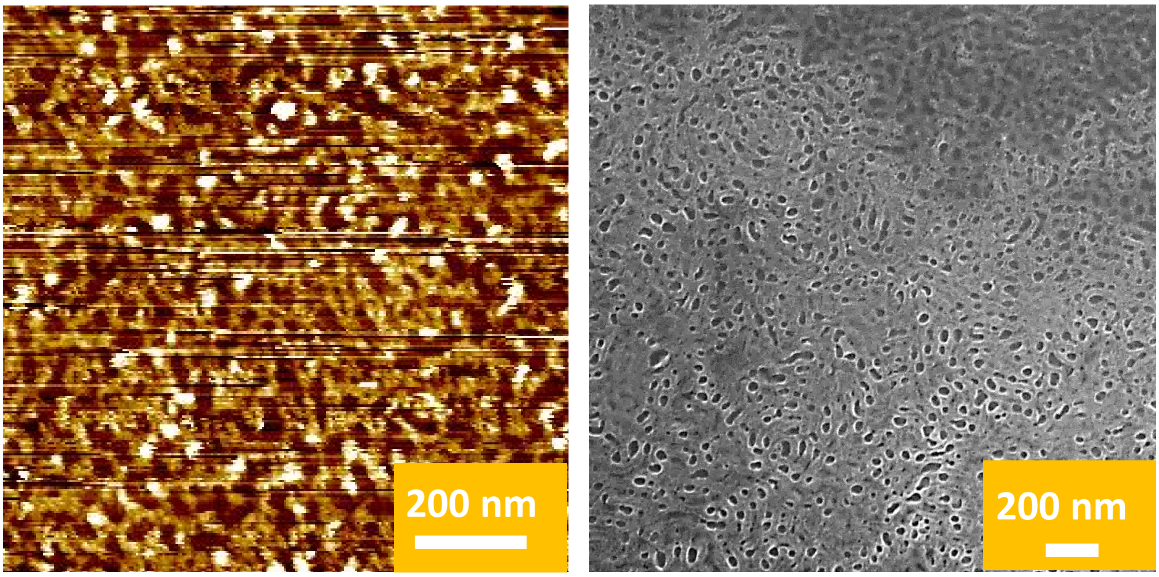

Figure S3 Topographical AFM(a), and SEM(b) images of mixed Ga and In oxide after UVO treatment.

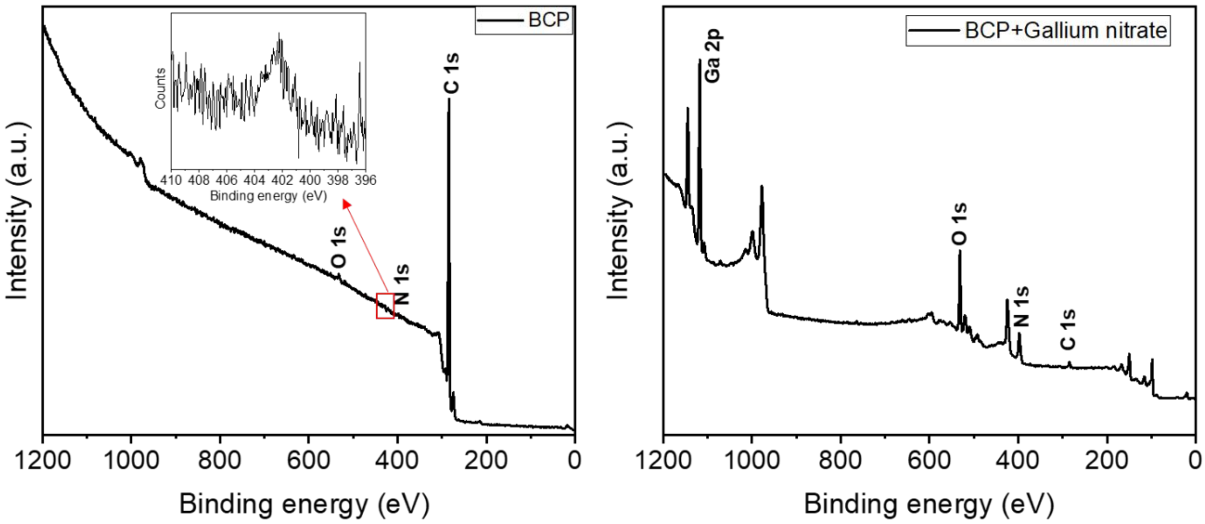

Figure S4 XPS spectra for (a) BCP, and (b) BCP+Gallium nitrate. The BCP template used here was formed after 20 hours of solvent annealing.

Table S3: Fitting parameters and obtained values for Ga 2p used in this study.

Name	Position	FWHM	L.Sh.	Area	%Area
Ga 2p	1118.16	2.26	GL (20)	12187.45	66.67
Ga 2p	1144.94	2.18	GL (20)	6093.72	33.33

References

(1) Flory, P. *Principles of Polymer Chemistry*; Cornell University Press: New York, 1953.

(2) Lindvig, T.; Michelsen, M. L.; Kontogeorgis, G. M. A Flory-Huggins Model Based on the Hansen Solubility Parameters. *Fluid Phase Equilib.* **2002**, *203* (1–2), 247–260. https://doi.org/10.1016/S0378-3812(02)00184-X.

(3) Hansen, C. M. *Hansen Solubility Parameters*, 2nd ed.; CRC Press, 2007. https://doi.org/10.1201/9781420006834.

(4) Venkatram, S.; Kim, C.; Chandrasekaran, A.; Ramprasad, R., Critical Assessment of the Hildebrand and Hansen Solubility Parameters for Polymers. J Chem Inf Model 2019, 59 (10), 4188-4194

(5) Kumar L, Horechyy A, Bittrich E, Nandan B, Uhlmann P, Fery A. Amphiphilic Block Copolymer Micelles in Selective Solvents: The Effect of Solvent Selectivity on Micelle Formation. Polymers (Basel). 2019 Nov 14;11(11):1882. doi: 10.3390/polym11111882. PMID: 31739558; PMCID: PMC6918162.

(6) Saleem, S.; Rangou, S.; Abetz, C.; Filiz, V.; Abetz, V. Isoporous Membranes from Novel Polystyrene-*b*-poly(4-vinylpyridine)-*b*-poly(solketal methacrylate) (PS-*b*-P4VP-*b*-PSMA) Triblock Terpolymers and Their Post-Modification. *Polymers* **2020**, *12*, 41. https://doi.org/10.3390/polym12010041

(7) Stull, Daniel R., *Vapor Pressure of Pure Substances. Organic and Inorganic Compounds*, **Ind. Eng. Chem.**, 1947, 39, 4, 517-540, https://doi.org/10.1021/ie50448a022 .
